# The importance of CFTR expression for neutrophil function in patients with Cystic Fibrosis

**DOI:** 10.1186/1753-6561-9-S1-A36

**Published:** 2015-01-14

**Authors:** B Jundi, K Pohl, McElvaney, E Reeves

**Affiliations:** 1 Department of Medicine, Respiratory Research, Beaumont Hospital, Dublin 9, Ireland

## Background

Cystic fibrosis (CF) is a genetic disease characterised by chronic bacterial infection of the lung and destruction of lung tissue eventually leading to respiratory failure. CF is caused by mutations in the CF transmembrane conductance regulator (CFTR) gene. Current treatment focuses on managing the symptoms of CF including antibiotic therapy against respiratory infections and vitamin and enzyme supplements to treat pancreatic insufficiency. However, a new drug known as ivacaftor has been approved recently that treats the underlying defect and corrects the defective CFTR in carriers of the G551D mutation. Neutrophils in CF fail to eradicate pathogens causing lung infections. Reports suggesting dysregulated neutrophil activity in CF illustrated altered gene expression and increased release of proteases from primary granules. However, it remains unknown whether neutrophil dysfunction is due to chronic inflammation or the CFTR defect. Our hypothesis is that impaired neutrophil activity in CF is directly caused by a lack of CFTR protein and function. Therefore, the aim of this study was to examine CFTR expression in neutrophils by optimising the methods for optimal CFTR protein detection, by comparing the levels of expression of mature CFTR protein in healthy control and CF neutrophils to epithelial cells and by examining the function of the CFTR channel in neutrophils.

## Methods

Ethical approval was obtained from Beaumont Hospital institutional review board. Cell proteins were isolated from purified neutrophils from healthy controls and CF patients. The CFTR protein was investigated by Western blot analysis. Healthy control cells were loaded with the chloride sensitive dye MQAE and changes in intracellular chloride were measured following treatment with the CFTR inhibitor CFTR(inh)-172 (10 mM) to examine the function of the channel.

## Results

Results clearly confirm the expression of the CFTR channel in neutrophils with levels of the mature, membrane CFTR being reduced in CF cells. Inhibition of CFTR function using the CFTR(inh)-172 resulted in accumulation of cytosolic chloride in healthy neutrophils.

## Conclusions

The results of this study strongly support a role for CFTR in neutrophil activity and dysfunctional CFTR may directly cause the impaired neutrophil killing ability which is observed in CF patients. Additionally, the presence of the CFTR protein makes it possible to treat neutrophil dysfunction directly using new drugs such as ivacaftor that correct the CFTR defect. This study was funded by the Alumni Office Claire and Nid Afdhal Award in Medicine.

